# Rare anatomic variation: Nasopharyngeal middle turbinate

**DOI:** 10.1002/ccr3.2344

**Published:** 2019-08-02

**Authors:** Jérôme R. Lechien, Alexandra Rodriguez, Stelianos Kampouridis, Mihaela Horoi

**Affiliations:** ^1^ Department of Otolaryngology and Head and Neck Surgery, CHU de Bruxelles, CHU Saint‐Pierre, School of Medicine Université Libre de Bruxelles Brussels Belgium; ^2^ Laboratory of Anatomy and Cell Biology, Faculty of Medicine, Research Institute for Health Sciences and Technology University of Mons (UMONS) Mons Belgium; ^3^ Department of Radiology, CHU de Bruxelles, CHU Saint‐Pierre, School of Medicine Université Libre de Bruxelles Brussels Belgium

**Keywords:** anomaly, development, nasal turbinate, nasopharynx, rhinosinusitis

## Abstract

Nasopharyngeal middle turbinate may be not systematically associated with recurrent chronic rhinosinusitis and could be indolent.

## 1 INTRODUCTION

The nasopharyngeal development of middle turbinate is a very rare nasal anatomic variation that has been suggested as anatomic favoring factor of recurrent rhinosinusitis, requiring sinus surgery. We report an unusual case of unilateral nasopharyngeal middle turbinate in a patient with rhinosinusitis who was successfully treated with medical treatment.

Nasal turbinates are anatomical structures extended from the lateral nasal wall into the nasal cavity. Embryologically, the ethmoid bone originates from the prechordal plate. At the 7 week of the embryonic development, three ridges develop lying in an antero‐posterior direction in the lateral nasal wall, which are smooth until this period. The inferior, middle, and superior nasal turbinates develop from these ridges. During the developmental process of turbinates, some anatomical variations may occur, the most notable being the pneumatization of the middle nasal turbinate (concha bullosa). Other anatomical variations of the middle nasal turbinate include the paradoxical middle turbinate, bifid or trifid middle turbinate, secondary middle turbinate, trifurcate middle turbinate, middle turbinate hypogenesis or agenesis.^1^, ^2^, ^3^ The identification of these nasal anatomical variations is relevant in case of functional endoscopic sinus surgery and because some of them are suspected to be associated with the development of chronic rhinosinusitis (CRS).^3^ In this paper, we report a very rare case of unilateral nasopharyngeal middle turbinate in a patient with CRS. The potential role of this anatomical variation in the development of CRS is discussed.

## 2 CASE REPORT

**Figure 1:**
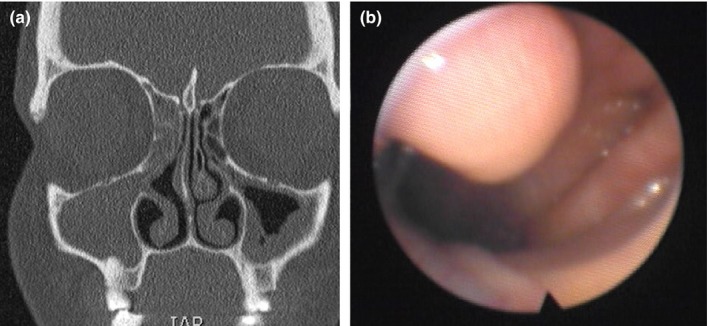
CT Scan and nasofibroscopy reporting posterior middle turbinate. The coronal plan of CT Scan showed a chronic rhinosinusitis of both maxillary sinuses A, The nasofibroscopy of the left nasal cavity exhibited a mass in the nasopharyngeal cavity.

**Figure 2:**
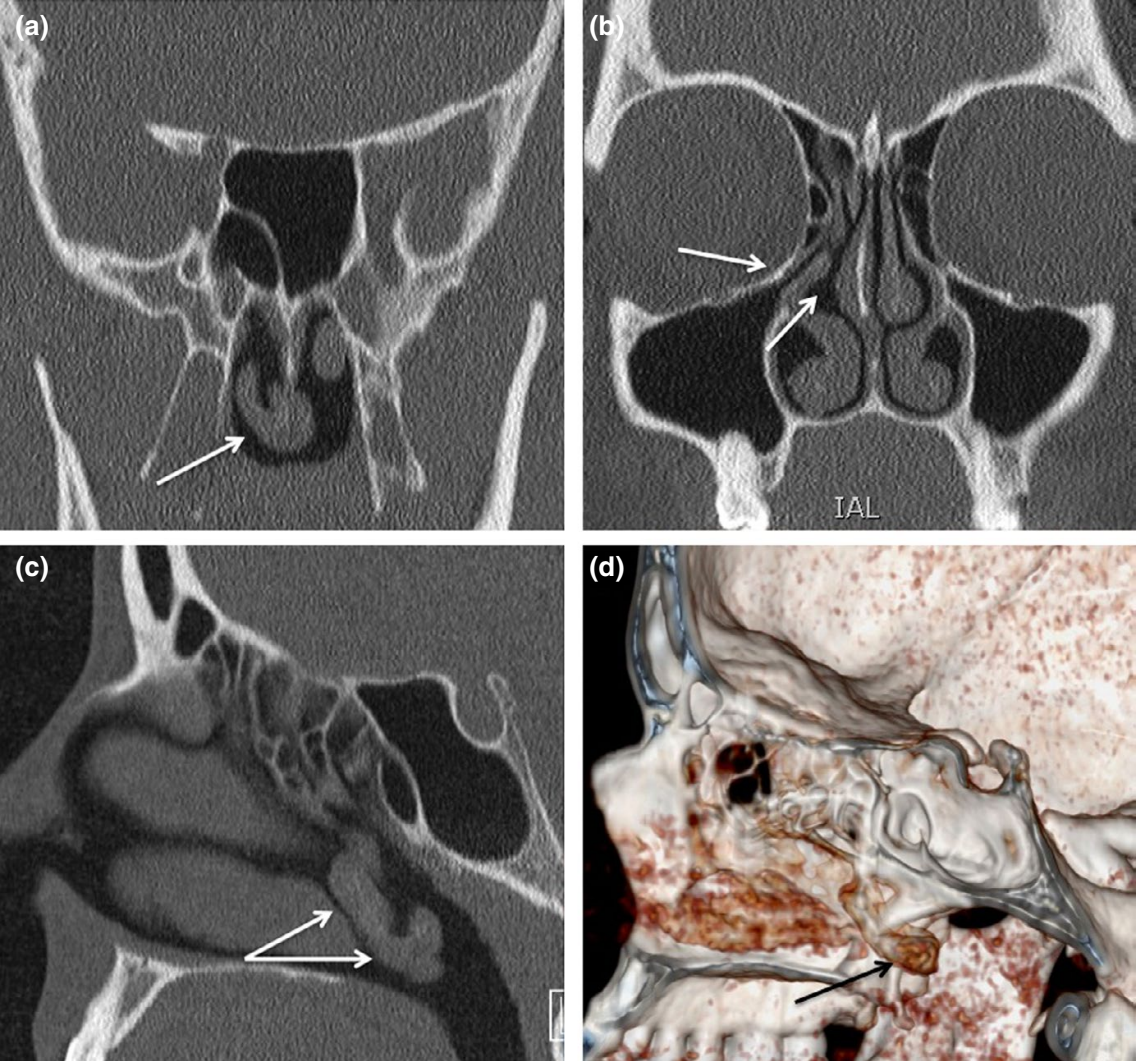
CT Scan reporting the anatomy of the posterior middle turbinate. The coronal (A, B), sagittal C, and 3D reconstruction D, showed the posterior development of the right middle turbinate. The anatomy of uncinate process and right middle meatus was normal B.

A 35‐year‐old woman was referred to the Department of Otolaryngology—Head and Neck Surgery for a first episode of a 6‐month history of right nasal obstruction, anterior rhinorrhea, postnasal drip and sneezing. The nasofibroscopy of the left nasal fossae showed an ovoid mass located in the nasopharynx (Figure 1). The examination of the right side did not see the right middle turbinate but identified a pedunculated hard mass that was extended from the lateral nasal wall (middle third) to the nasopharyngeal cavity. The mass was covered by nasal mucosa. Patient did not have history of head and neck surgery or traumatism, or allergy (negative skin test). Cardiovascular, respiratory, abdominal, and neurological examinations were unremarkable. The head and neck CT Scan reported a bilateral maxillary rhinosinusitis with a right total maxillary filling and the presence of a long pedunculated bony and mucosal structure extended from the medial bony wall of the right maxillary sinus to the nasopharyngeal cavity (Figures 1, 2). According to both clinical findings and CT Scan, the structure was suspected to be an abnormal middle turbinate. A medical treatment (clarithromycin, topic corticosteroids, and nasal saline solution) was administrated for a period of 3 months. At the end of the treatment, patient had a significant reduction of anterior rhinorrhea, postnasal drip, sneezing, findings, and the CT Scan showed a complete resolution of the disease. The right nasal obstruction persisted after treatment, especially in a supine position. There were no other comorbidities (laryngopharyngeal reflux, etc) explaining the remaining nasal obstruction. The follow‐up period (1 year) was unremarkable.

## 3 DISCUSSION

Chronic rhinosinusitis (CRS) is a common disease in United States concerning 14% of the population.^4^ The involvement of anatomical malformation in the development of CRS is still controversial for many decades. Overall, the anatomical malformations that have been suggested to be associated with an increased risk of CRS are septal deviation,^5^ concha bullosa,^6^ paradoxical middle turbinate,^7^ and teeth protrusion in the maxillary sinus.^8^ The role of other anatomical malformations was less studied according to the few reported cases. To date, only one case of posterior development of middle turbinate was reported and authors suggested that this abnormality could be an additional anatomical risk factor of recurrent CRS.^9^ Furthermore, they needed to treat this patient with functional endoscopic sinus surgery and the resection of the abnormal turbinate. In the present case, the significant improvements of complaints, endoscopic findings, and CT Scan, as well as the unremarkable follow‐up, do not support this association between nasopharyngeal middle turbinate and recurrent CRS. Moreover, as reported in Figure 2, the posterior development of middle turbinate was not associated with anatomical abnormalities of the middle meatus, uncinate process, and lateral wall of the right nasal cavity. Theoretically, the occurrence of an abnormality of one of these anatomical structures would have explained a believable risk factor of recurrence of CRS.

However, although a total resolution of both symptoms and findings, our patient had persistent posterior nasal obstruction, especially in supine. With regard to the position of the back of the middle turbinate in the inferior part of the choanal orifice, it is highly probable that this anomaly is associated with recurrent posterior nasal obstruction that is increased with the blood congestion of the nasal turbinate related to the supine position.

To our knowledge, this case is the second reported case of a posterior development of middle turbinate. The prevalence of this kind of malformation is unknown and the occurrence of this anomaly in a patient with CRS could be a coincidence. Future morphological studies including a very large number of healthy subjects and patients with CRS could specify a potential association between posterior development of middle turbinate, recurrent CRS, and recurrent posterior nasal congestion.

## CONFLICT OF INTEREST

None declared.

## AUTHORS' CONTRIBUTIONS

JL: Wrote the paper, analyzed the case, conducted literature review, and performed the research. AR: Analyzed the case and wrote the part: “case report”. SK: Is the radiologist who provided the images relating to the case, he also corrected a large part of the paper (clinical arguments and English language). MH: Coordinated, read, and corrected the paper.
